# Quality of pediatric clinical practice guidelines

**DOI:** 10.1186/s12887-021-02693-1

**Published:** 2021-05-07

**Authors:** Yali Liu, Yuan Zhang, Shu Wang, Ling Liu, Gang Che, Jiahui Niu, Yuan Ma

**Affiliations:** 1 Center for Clinical Epidemiology and Evidence-based Medicine, Beijing Children’s Hospital, Capital Medical University, National Center for Children’s Health, No. 56 Nanlishi Road, Xicheng District, Beijing, 100045 China; 2 Dalian Medical University, Dalian, 116044 Liaoning China; 3 Department of Pediatric Hematology-Oncology, Dalian Municipal Women and Children’s Medical Center, Dalian, 116037 Liaoning China; 4 Department of Neurosurgery, SanBo Brain Hospital, Capital Medical University, Beijing, 100093 China; 5 The First Hospital of Lanzhou University, Lanzhou, 730000 Gansu China; 6 Capital Medical University, Beijing, 100069 China

**Keywords:** Quality, Pediatric, Clinical practice guidelines, Evidence-based medicine

## Abstract

**Background:**

There is a lack of a comprehensive evaluation for pediatric clinical practice guidelines (CPGs) published in recent years. Here, we assessed the quality of pediatric CPGs, considering factors that might affect their quality. The aim of the study is to promote a more coherent development and application of CPGs.

**Methods:**

Pediatric CPGs published in PubMed, MedLive, Guidelines International Network, National Institute for Health and Care Excellence, and World Health Organization between 2017 and 2019 were searched and collected. Paired researchers conducted screening, data extraction, and quality assessment using the Appraisal of Guidelines for Research and Evaluation II (AGREE II). Linear regression analysis determined the factors affecting CPGs’ quality.

**Results:**

The study included a total of 216 CPGs, which achieved a mean score of 4.26 out of 7 points (60.86%) in the AGREE II assessment. Only 6.48% of the CPGs reached the “recommend” level. The remaining 69.91% should have been modified before recommendation, while the other 23.61% did not reach the recommended level at all. The overall quality of recent pediatric CPGs was higher than previously, and the proportion of CPGs with low-quality decreased over time. However, there were still too few CPGs that reached a high-quality level. The “applicability” and “rigor of development” domains had generally low scores. CPGs formulated by developing countries or regions, those that are not under an organizations or groups responsibility, and those that used non-evidence-based methods were found to be associated with poorer quality in different domains as independent or combinational factors.

**Conclusions:**

The quality of pediatric CPGs still needs to be improved. Specifically, a quality control before applying new CPGs should be essential to ensure their quality and applicability.

**Supplementary Information:**

The online version contains supplementary material available at 10.1186/s12887-021-02693-1.

## Background

Clinical practice guidelines (CPGs) are statements to guide health providers and patients [1]. High-quality and rigorously-developed CPGs with appropriate recommendations improve clinical and public health outcomes by helping health providers follow the right clinical practice [2, 3]. Furthermore, policymakers and educators can establish more appropriate health policies and enhance appraisal skills in education with the help of CPGs [4, 5]. However, implementation of CPGs with insufficient quality or inappropriate contents may mislead clinicians [6, 7]. Therefore, it is essential to develop CPGs with better quality and appropriate content. When implementing CPGs in everyday clinical practice, users should pay attention to the content and local adaptations of the guidelines and their quality [8, 9].

The Appraisal of Guidelines for Research and Evaluation (AGREE) instrument was first proposed in 2003 to verify the quality of CPGs by the AGREE collaboration [10]. After that, the updated AGREE II [11] and a checklist, Reporting Items for Practice Guidelines in Healthcare (RIGHT) [12], were released. Although AGREE II has several limitations, especially related to the assessment of CPGs content [13–15], it is still a helpful and widely recognized tool for assessing CPG quality [16, 17]. AGREE II can also provide a methodological strategy in CPG development, which is very useful for CPG developers, health care providers, policymakers, and educators [18]. So far, it has been widely used and recognized in the quality assessment of CPGs [16, 17].

Recently, the number of pediatric CPGs grew substantially. However, some reports raised concerns about their quality [19, 20]. Previous quality assessments of pediatric CPGs are out of date [21, 22] or only focus on a certain field [23, 24]. A comprehensive and up-to-date evaluation of the quality of pediatric CPGs published in recent years is lacking [25–27]. Therefore, the present study aimed to systematically search pediatric CPGs published between 2017 and 2019, assess their quality, and explore the factors that might influence them.

## Methods

### Eligibility criteria

To be included in the study, CPGs had to be either clinical practice guidelines, clinical treatment guidelines, or clinical recommendations focused on the pediatric population, defined as under 18 years old or a subset of it. All included CPGs should be in English to represent internationally recognized CPGs. The present study aimed to evaluate recent CPGs; therefore, we only included pediatric CPGs published between 2017 and 2019. We excluded documents that were not original CPGs (i.e., literature reviews, position papers, letters; paraphrase, interpretation, or analysis of previous CPGs). We included only the newest revised version of CPG updates published between 2017 and 2019, to prevent multiple counting.

### Search strategy

The following search engines and databases were systematically searched, PubMed (pubmed.gov), MedLive (guide.medlive.cn), Guidelines International Network (GIN; g-i-n.net), National Institute for Health and Care Excellence (NICE; nice.org.uk), and World Health Organization (WHO; who.int). The language limit was set as “English” and the published time limit was “from January 1, 2017, to December 31, 2019”. The searching terms included pediatric restriction, “Child (M, for Mesh)” or “Child, Preschool (M)” or “Infant (M)” or “Adolescent (M)” or “Infant, Newborn (M)” or “Child* (* for wildcard)” or “pediat*” or “paediat*” or “infan*” or “youth*” or “toddler*” or “adolesc*” or “teen*” or “boy*” or “girl*” or “bab*” or “preschool*” or “pre-school*”; and guideline restriction, “Practice Guideline (Publication Type) or “Guideline*” or “Guidance*” or “Recommendation*” or “Consensus*.”

### Guideline selection and data extraction

The CPG selection and data extraction procedures were accomplished by two researchers independently. After cross-checking the selected CPGs and extracting data, the two researchers reached a consensus. In case disagreements occurred, an experienced senior reviewer was consulted and made the final decision.

**Fig. 1:**
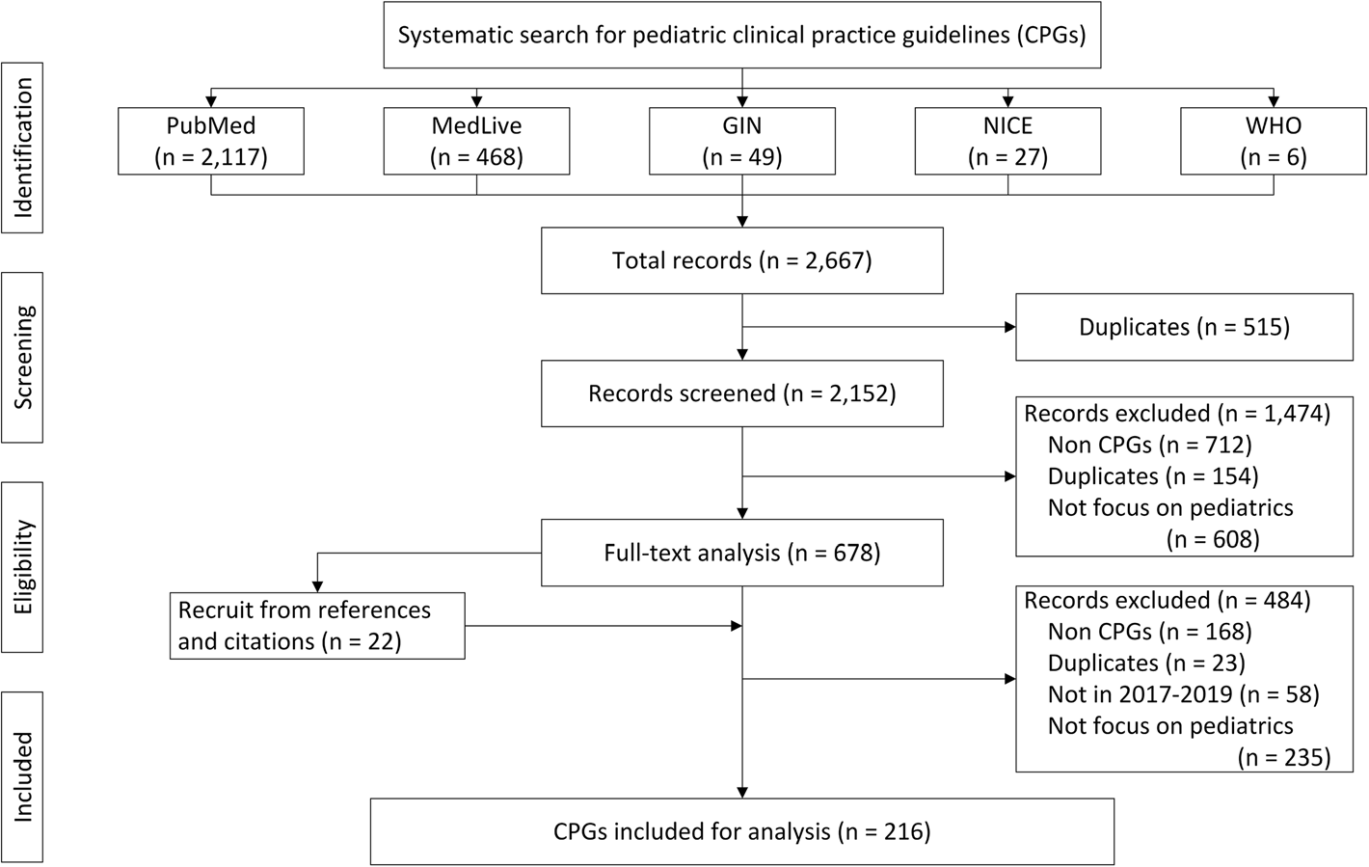
Flow diagram of the systematic searching and selecting for pediatric clinical practice guidelines (CPGs) procedure. CPGs: Clinical practice guidelines; GIN: Guidelines International Network; NICE: National Institute for Health and Care Excellence; WHO: World Health Organization

After summarizing the records from all databases, we ran a software-assisted (Endnote; Clarivate Analytics, MA., USA, version 20) [28] duplication process on the data set, followed by a two-step selection procedure. The first step was to select CPGs that potentially met the eligibility criteria by screening titles and abstracts. After that, a full-text analysis determines the CPGs to include in the final data set. To prevent omissions, a group of researchers was arranged to search for CPGs from references and citations of previously included CPGs. Figure 1 shows the systematic searching and selection procedure.

The data extraction procedure collected the following parameters: published year, country or region of origin (divided into developing and developed countries or regions according to the list of World Trade Organization (WTO), version 2019), organization or group responsible for CPG development (individual, few persons, or small teams were excluded), applied population, and field of focus (based on the International Classification of Diseases 11th Revision, ICD-11; released by WHO on June 18, 2018). After reviewing the full text, the reviewers also assessed whether the methodology and CPGs were evidence-based or not. The evidence-based CPGs were defined by the Health and Medicine Division of the American National Academies as “statements that include recommendations intended to optimize patient care and are informed by a systematic review of evidence and an assessment of the benefits and harms of alternative care options” [29]. Evidence-based CPGs needed to be based on summarizing and analyzing existing evidence. The other CPGs that lacked evidence-base (e.g., based on expert opinion only) were considered as non-evidence-based CPGs.

### Quality assessment

The quality of the included CPGs was appraised by two reviewers using the AGREE II instrument [18]. The reviewers were pediatricians who had extensive clinical pediatrics and evidence-based medicine experience. Before the appraisals, the reviewers completed AGREE II online tutorial training (agreetrust.org) and practiced under the supervision of a senior experienced reviewer. A multi-round test assessment was required for the two reviewers. In the first round, each reviewer was required to independently assess ten randomly selected CPGs. The scores assigned by these two reviewers in each item were tested for consistency by the Intraclass Correlation Coefficient (ICC). For items that achieved ICC values less than 0.85, the reviewers needed to review the AGREE II instrument and discussed the discrepancy to reach a consensus. After that, another test assessment was conducted in the second round. The assessment was considered complete after we finished at least three-round tests and achieved an ICC value no less than 0.85 in each item.

The AGREE II consists of 23 key items in 6 domains to capture different dimensions of CPG quality, which include scope and purpose (items 1–3), stakeholder involvement (items 4–6), rigor of development (items 7–14), clarity of presentation (items 15–17), applicability (18–21), and editorial independence (items 22–23) [11, 18]. Each item is assigned a score from 1 (strongly disagree, when no given information is relevant) to 7 (strongly agree, when full criteria of the item are met). The more criteria that are met, the higher the scores are given. According to the AGREE II instrument, scores of each domain are calculated as follows: the difference between maximum possible score and minimum possible score divided by the difference between actually obtained score and minimum possible score. Furthermore, according to the instrument, the reviewers provided two overall assessments of the CPG based on the six domains’ quality. The reviewers assigned an overall quality score from 1 to 7 (higher scores indicating higher quality) by taking into account the total scores from each of the six domains as well as personal judgement made by the reviewers. If the overall assessment scores given by these two reviewers differed by 1 point, the lower score was assigned; if it varied by 2 points, the average scores were assigned; and if it differed by ≥3 points, the reviewers reviewed for agreement [17]. To reach the recommended level, CPGs had to achieved overall assessment scores of 6 and 7 (above 80% of 7 scores). With an overall score of 4 and 5 (60 to 80% of 7 scores) the level was “recommended with modification”, while CPGs with a score of 1 to 3 (less than 60% of 7 scores) were not recommend [19, 30, 31]. Taking into account the criteria considered in the assessment process, if the CPG had serious issues in one of the domains, it would be downgraded one level [17, 31].

To ensure the validity and reliability of the assessment, after the overall assessment procedure, 10% of the assessments were randomly selected by a senior experienced reviewer and re-assessed. The samples were divided with simple random sampling, and the random number table was generated by the SPSS software pack (IBM, NY, USA; version 26). Additionally, the overall quality scores of CPGs in different fields, organizations, groups, countries, or regions were summarized and ranked. Only variables with at least 3 CPGs were given a ranking. The ranking was based on the mean overall assessment scores.

### Statistical analysis

Continuous variables (e.g., AGREE II scores) were presented as mean; categorical variables (e.g., recommendation levels) were reported as a number and a percentage. The comparison of categorical variables was conducted by Pearson’s *x*^*2*^ test or Fisher’s exact test as appropriate. The two groups’ continuous variables were compared using a two-sample *t*-test or Mann-Whitney signed-rank test determined by data distribution and variance homogeneity. The Kolmogorov-Smirnov test was used as a normal distribution test. Leneve’s test was conducted to explore the homogeneity of variance. The association between appraised scores and the characteristics of CPGs was analyzed by the linear regression to explore potential influential factors of CPGs’ quality. The independent variables were set as country or region development (developing or developed), organization or group responsible (yes or no), and evidence-based method (yes or no). A *p*-value < 0.05 was considered significant. All statistical analyses were performed with SPSS software pack (IBM, NY, USA; version 26).

## Results

### Guideline selection and characteristics

Overall, the search identified 2667 records, and 515 records were deleted in the software-assisted duplicates elimination process [28]. In the screening process, 1474 records were excluded (712 records were not CPGs, 154 records were duplicates, and 608 records did not focus on pediatrics). After including 22 records from references and citations and after excluding 484 records (168 records were not CPGs, 23 records were duplicates, 58 records did not publish in 2017 to 2019, and 235 records did not focus on pediatrics) in the full-text analysis, a total of 216 pediatric CPGs were used. Detailed selection procedures are shown in Fig. 1.

Among these CPGs, 71.3% were compiled by developed countries or regions; 85.65% of them were through organizations or groups. Three-quarters of included CPGs used evidence-based methods to develop CPGs, while the other one quarter did not. Table 1. shows the characteristics of included pediatric CPGs.

**Table 1:** Characteristics of included pediatric clinical practice guidelines (*n* = 216)

Subject	n	%
Country/ Region		
Developed	154	71.30
Developing	62	28.70
Organization/ Group		
Yes	185	85.65
No	31	14.35
Evidence based		
Yes	162	75.00
No	54	25.00
Field (ICD-11 code)		
1 Infectious	5	2.31
2 Neoplasms	16	7.41
3 Blood	3	1.39
4 Immune	4	1.85
5 Endocrine	36	16.67
6 Mental	14	6.48
7 Sleep	1	0.46
8 Nervous	12	5.56
9 Visual	1	0.46
10 Ear	6	2.78
11 Circulatory	7	3.24
12 Respiratory	11	5.09
13 Digestive	10	4.63
14 Skin	1	0.46
15 Musculoskeletal	11	5.09
16 Genitourinary	12	5.56
17 Sexual	3	1.39
19 Perinatal	33	15.28
20 Developmental	7	3.24
21 Symptoms	6	2.78
22 External	1	0.46
-^a^ General	16	7.41

^a^General fields (e.g., screening and diagnosis)

### Quality assessment

**Fig. 2:**
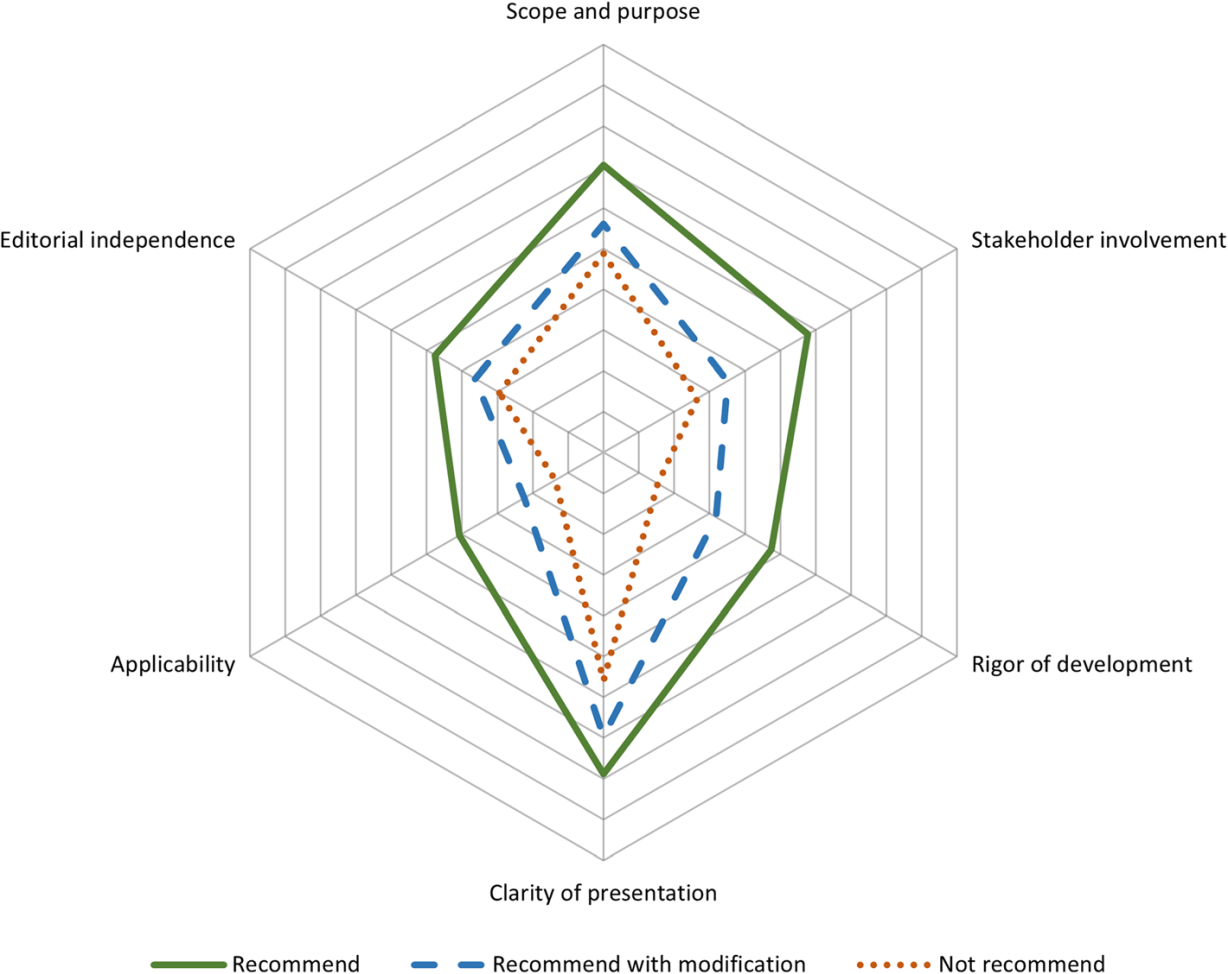
Summary of standardized scores in each domain of different recommendation level guidelines by AGREE II. Continuous variable (standardized scores) is presented as mean (%); AGREE II: The Appraisal of Guidelines for Research and Evaluation II

The included CPGs achieved a mean score of 4.26 out of 7 points (60.86%) in the overall AGREE II assessment. Only 6.48% of the CPGs reached the “recommend” level, 69.91% needed modifications before reaching the “recommend” level, and the other 23.61% CPGs were not recommended. In the six domains assessment, the “clarity of presentation” domain achieved the highest mean score of 66.77%. The “applicability” domain had the poorest mean quality, only achieving a mean score of 21.26%. CPGs compiled by developed countries or regions and under organizations or groups achieved higher scores in different domains. Evidence-based CPGs achieved a significantly higher score lead in nearly all domains, overall assessment scores (*p* < 0.001), and recommendation levels (*p* < 0.001) compared to non-evidenced CPGs. The score of overall CPGs and subgroups are presented in Table 2. The scores in each domain of different recommendation levels are summarized in Fig. 2. The CPGs that achieved lower recommendation levels were insufficient in “applicability” and “rigor of development”.

**Table 2:** Comparison of standardized scores in each domain of guidelines and subgroups by AGREE II

Subject	Scope and purpose ^a^	Stakeholder involvement ^a^	Rigor of development ^a^	Clarity of presentation ^a^	Applicability ^a^	Editorial independence ^a^	Overall assessment ^a^	Recommend ^b^	Recommend with modification ^b^	Not recommend ^b^
Overall	55.16%	34.22%	28.62%	66.77%	21.26%	35.26%	4.26	6.48%	69.91%	23.61%
Country/ Region										
Developed	57.59%	35.79%	28.70%	68.32%	22.85%	38.37%	4.78	6.25%	77.08%	16.67%
Developing	52.11%	32.26%	28.53%	65.53%	19.27%	31.38%	4.12	5.00%	64.17%	30.83%
*p*	0.001^**^	0.080	0.943	0.144	0.060	0.023^*^	0.052	–	–	0.055
Organization/ Group										
Yes	55.67%	34.32%	28.98%	68.35%	22.26%	34.93%	4.36	6.49%	72.43%	21.08%
No	52.15%	33.60%	26.51%	57.35%	15.26%	37.23%	3.65	3.23%	54.84%	41.93%
*p*	0.083	0.801	0.470	< 0.001^**^	< 0.001^**^	0.595	< 0.001^**^	–	–	0.009^†^
Evidence based										
Yes	56.41%	35.75%	31.88%	69.00%	22.70%	35.98%	4.48	7.41%	75.31%	17.28%
No	51.39%	29.63%	18.85%	60.08%	16.94%	33.10%	3.59	1.85%	53.70%	44.45%
*p*	0.008^**^	< 0.001^**^	< 0.001^**^	< 0.001^**^	0.001^**^	0.358	< 0.001^**^	–	–	< 0.001^†^

*AGREE II* The Appraisal of Guidelines for Research and Evaluation II; ^a^ Continuous variable is presented as mean; ^b^ Categorical variable is reported as percentage; ^*^p < 0.05 (two-sample *t*-test); ^**^p < 0.01 (two-sample *t*-test); ^†^*p* < 0.01 (Person’s *x*^*2*^ test)

Additionally, the score of CPGs in different fields (Supplemental Table 1.), organizations or groups (Supplemental Table 2.), and countries or regions (Supplemental Table 3.) were summarized and ranked. The CPGs related to the circulatory system, digestive system, and general fields (e.g., screening and diagnosis) achieved higher overall assessment scores. The CPGs developed under the WHO, Queensland Health (QH), and the American Academy of Pediatrics (AAP) responsibility had the highest quality. For different countries or regions’ comparisons, CPGs developed by the U.K., Australia, and Italy had better quality.

### Influential factors

The multi-factor linear regression was used to explore the association between scores in each domain and the characteristics of CPGs. After analysis, CPGs which were not organization or group responsible (β = − 0.179; 95% CI = − 1.017, − 0.175; *p* = 0.006) and those that used a non-evidence-based method (β = − 0.312; 95% CI = − 1.180, − 0.498; *p* < 0.001) were associated with poorer overall quality. Furthermore, CPGs formulated by developing countries or regions, those that are not under an organizations or groups responsibility, and those that used non-evidence-based methods were found to be associated with poorer quality in different domains as independent or combinational factors, as shown in Table 3.

**Table 3:** Association of standardized scores in each domain by AGREE II and characteristics of guidelines using linear regression

Domain and subject	*R*^2^	Adjusted *R*^2^	β	95% CI	*p*
Scope and purpose	0.101	0.089			
Constant			–	0.573, 0.620	< 0.001^**^
Country/ Region			− 0.251	− 0.092, − 0.029	< 0.001^**^
Organization/ Group			−0.096	− 0.077, 0.011	0.144
Evidence based			− 0.191	− 0.089, − 0.017	0.004^**^
Stakeholder involvement	0.051	0.038			
Constant			–	0.347, 0.406	< 0.001^**^
Country/ Region			−0.136	−0.079, − 0.001	0.044^*^
Organization/ Group			−0.002	−0.057, 0.055	0.972
Evidence based			−0.192	−0.110, − 0.020	0.005^**^
Rigor of development	0.106	0.093			
Constant			–	0.290, 0.360	< 0.001^**^
Country/ Region			−0.034	−0.057, 0.033	0.608
Organization/ Group			−0.010	−0.069, 0.060	0.881
Evidence based			−0.325	−0.183, − 0.079	< 0.001^**^
Clarity of presentation	0.141	0.129			
Constant			–	0.666, 0.720	< 0.001^**^
Country/ Region			0.061	−0.018, 0.052	0.341
Organization/ Group			−0.242	−0.146, − 0.046	< 0.001^**^
Evidence based			−0.242	−0.118, − 0.037	< 0.001^**^
Applicability	0.081	0.068			
Constant			–	0.227, 0.283	< 0.001^**^
Country/ Region			−0.156	−0.080, − 0.007	0.020^*^
Organization/ Group			−0.167	−0.118, − 0.014	0.013^*^
Evidence based			−0.172	−0.097, − 0.013	0.013^*^
Editorial independence	0.031	0.017			
Constant			–	0.345, 0.437	< 0.001^**^
Country/ Region			−0.160	−0.131, − 0.012	0.019^*^
Organization/ Group			0.034	−0.064, 0.107	0.618
Evidence based			−0.074	−0.107, 0.031	0.278
Overall assessment	0.142	0.130			
Constant			–	4.386, 4.838	< 0.001^**^
Country/ Region			−0.055	−0.425, 0.166	0.389
Organization/ Group			−0.179	−1.017, − 0.175	0.006^**^
Evidence based			−0.312	−1.180, − 0.498	< 0.001^**^

*AGREE II* The Appraisal of Guidelines for Research and Evaluation II; ^*^p < 0.05 (linear regression); ^*^p < 0.01 (linear regression)

## Discussion

### Overall guideline quality

Previous studies assessing quality assessment of pediatric CPGs are outdated or only focused on a specific field [21–24]. Isaac et al. conducted a study in 2011 to evaluate the quality of development and reporting of 28 CPGs developed or endorsed by AAP. After assessment with AGREE II, they showed that the CPGs achieved an overall mean score of 55%, which is lower than the present study. Furthermore, they reported 29% of the CPGs with an overall score of < 50%, while this proportion decreased in the present study [21]. These results suggest that the overall quality of pediatric CPGs improved since 2011. However, the number of CPGs reaching high quality (receiving the “recommend” level) did not change significantly, compared with before [21]. Xie et al. appraised pediatric CPGs related to community-acquired pneumonia published from January 2000 to March 2015. In their study, 30% of CPGs achieved the “recommended” levels, 40% of CPGs were “recommended with modifications”, and 30% of CPGs were “not recommended” [32]. Generally, based on existing research, the overall quality of pediatric CPGs improved compared to early CPGs [21, 32]. However, there were still few CPGs that reached a high-quality level. Moreover, the overall quality score was still inadequately compared to the quality evaluation for other recent CPGs focused on adults. Most of the studies that focused on adult CPGs reported a mean overall AGREE II scores of 4.77–5.97 in 7 points (68.21–85.35%), and 8.2–50.0% of them could reach the “recommend” level [33–35]. A study published in 2018 analyzed 89 CPGs on adult critical care, and reported a mean overall score of 83%, which was higher than this review [36]. The study by Madera et al. suggested that 50% of the eight adult CPGs on screening and diagnosis of oral cancer were assessed as “recommend” and the other 50% were assigned as “recommended with modifications” [37]. Compared with CPGs for adults, the quality of pediatric CPGs still needs to be improved.

### Quality of domains

Compared with other studies using the AGREE II assessment, the present study also revealed that “applicability” and “rigor of development” domains had poorer quality [21, 22, 35, 36]. A study of previous assessment of pediatric CPGs showed that “applicability”, “editorial independence”, and “stakeholder involvement” domains achieved the lowest mean scores, at 19, 40, and 42%, respectively [22]. We also compared the scores of each domain among CPGs with different recommendation levels to determine which domains affect the recommendation level. As shown in Fig. 2, the CPGs that achieved lower recommendation levels were insufficient in “applicability” and “rigor of development”, which indicated these domains affected the overall quality of pediatric CPGs.

The “applicability” domain mainly focuses on the barriers and facilitators to apply the CPG [18]. This domain required CPGs to consider facilitators and barriers in the application, and provide advice or tools for different age groups and regions. The clinical manifestations, progress, and outcomes of pediatric diseases are different from those of adult diseases. Therefore, before applying a CPG, it is necessary to evaluate its quality and scope of application. The study of Boluyt et al. was a great example of adopting CPGs [22]. They conducted a systematic review of CPGs and assessed the quality and applicability of the CPGs. Furthermore, they synthesized the expert opinions to determine the CPGs that can be used in local clinical practice [22].

The “rigor of development” domain is the key to the development of a qualified CPG. This domain relates to gathering and synthesizing the evidence, promoting recommendations and update schedules of CPGs [18]. The AGREE II manual [11] and RIGHT checklist [12] provide various suggestions in CPG development and reporting, such as systematic methods, evidence criteria, review procedure, and update schedule, which should be consulted and followed in the proposal, development, report, review, and update procedures of a CPG.

Recently, several studies raised the concern that conflict of interest could affect the quality of CPGs [38–41]. However, only limited CPGs described the management of financial conflicts of interest [40]. Komesaroff et al. proposed the concept of “conflicts of interests” as “the condition that arises when two coexisting interests directly conflict with each other: that is when they are likely to compel contrary and incompatible outcomes” [39]; while Grundy et al. and Wiersma et al. suggested “non-financial conflicts of interests” should also receive awareness in health and medicine [41, 42]. The AGREE II provides a domain as “editorial independence” to evaluate whether the funding bodies have influenced the content and whether conflicts of interests of CPG development group members have been recorded and addressed [18]. Our study showed that the “editorial independence” domain achieved a mean score of only 35.26% for pediatric CPGs. In addition, several previous studies highlighted that “editorial independence” domain of AGREE II in pediatric CPGs had inappropriate quality (a mean score of 17–48%) [19–21]. Thus, the potential conflicts of interests in CPG development should be disclosed and reviewed carefully. Independent committees should also be engaged for evaluation and management [18, 40].

### Influential factors of quality

Some studies showed a significant improvement in CPGs’ quality under organizations or groups’ responsibility [8, 20]. According to the study of Font-Gonzalez et al., CPGs under organizations or groups’ responsibility were more likely to have high quality [20]. In the present study, only a few CPGs (14.3%) were not conducted by organizations or groups. Reliable organizations or groups can complete the CPG development procedures, use appropriate methods, and report in a more complete manner, which might be relatively difficult for an individual or small team [20]. Furthermore, a small team might lack the skills or training in developing CPGs as compared with large organizations or groups [20].

Previous studies suggested that a non-evidence-based method in CPG development might significantly affect quality [43]. In the present study, one-quarter of CPGs did not use evidence-based methods, and we found that non-evidence-based methods had significant influence in nearly all domains. The evidence-based method was important in CPG development and clinical decision-making [44]. By using an evidence-based method, we could systematically search and summarize previous research, reducing the limitations and bias [45].

Several studies suggested CPGs developed in regions with different economic development statuses might influent the quality of CPGs [22, 43]. The present study also found that CPGs developed by developing countries or regions had poorer quality in domains related to “scope and purpose”, “stakeholder involvement”, “applicability”, and “editorial independence”. Also, we found that most of the CPGs with poor quality developed by developing countries or regions did not follow a strict and comprehensive development procedure; and some of them did not use the evidenced-based method, which might influence quality. Most of the CPGs with high quality were developed by countries or organizations with significant funding and resource. A previous study suggested that AAP’s internal CPGs had significantly higher total scores than endorsed CPGs [21]. These CPGs with high quality were developed under a strictly completed, evidence-based CPG development procedure. Additionally, the CPG committee consisted of clinical experts, methodologists, and others involved from different fields, improving the rigor in development and applicability in practice [46]. For resource-limited developing countries, it might be a challenge to form a complete expert group to complete the CPG development procedure. One possible way of these regions was adapting existing high-quality CPGs [47]. In addition, international collaboration could be an acceptable way of developing a CPG [48]. However, as there were nuances in many healthcare systems worldwide that might preclude the direct deployment of international CPGs, agencies should consider CPG adaptations for their institutions. The process for guideline adaptation (ADAPTE) could create CPG versions, derived from existing CPGs, but modified to local settings, which is a cost-effective and less resource-intensive approach to CPG development [48]. Recently, Dizon et al. suggested a standardized procedure to adopt, adapt or contextualize recommendations from existing CPGs of good quality, promoting the use of scarce resources more focused on implementation [49]. These studies provided meaningful attempts at tailoring CPGs to the local context.

### Limitations

The present study had several limitations. Firstly, because the present study’s primary purpose was to evaluate the quality of recent pediatric CPGs, we only assessed CPGs published in the past 3 years, which limits the evaluation of the change in CPGs’ quality over time. Also, only English CPGs were included in this study; therefore, further research should analyze CPGs that were written in different languages when possible. Secondly, the AGREE II assessment was related to the personal judgment of reviewers, which might introduce selection bias. Thus, we conducted strict training and test assessment procedures. A re-assessment procedure was also performed to reduce selection bias. Finally, AGREE II has its inherent limitations. AGREE-II scores are dependent upon reporting, while some CPG committees may comply with the requirements but do not ultimately report. In addition, AGREE II only focuses on the quality in developing and reporting procedures of CPGs, but the evidence behind the recommendations cannot be evaluated. Thus, AGREE II is not sufficient to ensure that CPG recommendations are appropriate and accurate [13–15]. Several studies suggested that a new version of AGREE with an evaluation of CPGs’ contents should be proposed, which would require a great effort and collaboration [13–15]. We suggest health providers should closely follow new versions of well-developed tools for the appraisal of CPGs. Before that, health care providers should assess CPG quality using tools like AGREE II and evaluate CPG content and local adaptations before implying recommendations from a CPG [26, 50, 51]. Furthermore, different CPGs might contradict some recommendations, which cannot be solved by AGREE II alone. When these contradictions occur, health providers should review its contents and evidence. Thus, the decision to implement recommendations from CPGs requires careful considerations, including its quality, contents, adaptions, patients’ wishes, resources, feasibility, and fairness.

## Conclusions

In conclusion, the quality of the pediatric CPGs was rarely excellent. The overall quality of recent pediatric CPGs was higher than previous pediatric CPGs, and the proportion of CPGs with low quality decreased. However, there were still limited CPGs reaching a high-quality level. The “applicability” and “rigor of development” domains had low quality. CPGs formulated by developing countries or regions, those that are not under an organizations or groups responsibility, and those that used non-evidence-based methods were found to be associated with poorer quality in different domains as independent or combinational factors.

The quality of pediatric CPGs still needs more research and improvement. It is necessary to strengthen the development and reporting procedures of pediatric CPGs. Besides that, the quality and applicability of a CPG should be evaluated before its application.

## Supplementary Information

**Additional file 1: Supplemental Table 1**. Comparison of standardized scores in each domain of guidelines in different fields (ICD-11 code) by AGREE II.

**Additional file 2: Supplemental Table 2**. Comparison of standardized scores in each domain of guidelines established by different organizations or groups by AGREE II.

**Additional file 3: Supplemental Table 3**. Comparison of standardized scores in each domain of guidelines established by different countries or regions by AGREE II.

## Data Availability

All data generated or analyzed during this study are included in this published article and its supplementary information files.

## Abbreviations

CPG: Clinical practice guideline
AGREE: Appraisal of Guidelines for Research and Evaluation
RIGHT: Reporting Items for Practice Guidelines in Healthcare
PRISMA: Preferred Reporting Items for Systematic Reviews and Meta-analyses
GIN: Guidelines International Network
NICE: National Institute for Health and Care Excellence
WHO: World Health Organization
ASRM: American Society for Reproductive Medicine
QH: Queensland Health
AAP: American Academy of Pediatrics
