# Supplementary material for: Quality of pediatric clinical practice guidelines
Source: BMC Pediatr. 2021 May 7;21:223. doi: 10.1186/s12887-021-02693-1 (PMC8103635; doi:10.1186/s12887-021-02693-1)
Supplement: Supplementary file 1 — Additional file 1: Supplemental Table 1. Comparison of standardized scores in each domain of guidelines in different fields (ICD-11 code) by AGREE II. [file 12887_2021_2693_MOESM1_ESM.docx]

**Online Only**

Supplemental Table 1. Comparison of standardized scores in each domain of guidelines in different fields (ICD-11 code) by AGREE II.

| Subject | Scope and purpose | Stakeholder involvement | Rigor of development | Clarity of presentation | Applicability | Editorial independence | Overall assessment | Ranking ^a^ |
| --- | --- | --- | --- | --- | --- | --- | --- | --- |
| Overall | 55.16% | 34.22% | 28.62% | 66.77% | 21.26% | 35.26% | 4.26 | - |
| Field (ICD-11 code) | | |  |  |  |  |  |  |
| 1 | 56.67% | 26.67% | 27.08% | 67.22% | 21.67% | 30.00% | 4.00 | 18 |
| 2 | 54.51% | 33.16% | 30.40% | 66.84% | 19.27% | 43.75% | 4.06 | 17 |
| 3 | 56.48% | 34.26% | 28.82% | 61.11% | 36.80% | 43.05% | 4.33 | 6 |
| 4 | 54.86% | 40.28% | 33.07% | 59.72% | 20.32% | 58.34% | 4.25 | 9 |
| 5 | 44.44% | 26.00% | 21.24% | 72.53% | 15.74% | 16.67% | 4.08 | 14 |
| 6 | 55.36% | 39.88% | 23.74% | 59.52% | 26.93% | 30.95% | 4.50 | 5 |
| 8 | 57.64% | 42.13% | 34.38% | 59.96% | 21.18% | 47.22% | 4.08 | 15 |
| 10 | 60.65% | 35.65% | 31.25% | 71.30% | 17.36% | 27.78% | 4.17 | 12 |
| 11 | 57.14% | 38.10% | 35.57% | 70.24% | 21.43% | 48.21% | 4.86 | 1 |
| 12 | 57.58% | 33.33% | 29.55% | 60.86% | 20.08% | 34.85% | 4.18 | 11 |
| 13 | 59.72% | 32.50% | 45.52% | 66.67% | 27.92% | 47.08% | 4.60 | 2 |
| 15 | 52.02% | 37.12% | 35.99% | 60.35% | 20.65% | 35.23% | 4.55 | 4 |
| 16 | 57.41% | 34.49% | 35.68% | 71.53% | 19.97% | 32.64% | 4.08 | 16 |
| 17 | 66.67% | 51.85% | 29.51% | 62.96% | 30.56% | 45.83% | 4.33 | 7 |
| 19 | 61.28% | 34.09% | 23.93% | 70.87% | 25.25% | 42.43% | 4.24 | 10 |
| 20 | 56.75% | 39.28% | 17.71% | 60.71% | 16.37% | 25.59% | 4.29 | 8 |
| 21 | 51.39% | 31.02% | 31.95% | 66.67% | 16.32% | 48.61% | 4.17 | 13 |
| - ^b^ | 58.16% | 36.98% | 33.01% | 65.80% | 21.87% | 32.55% | 4.56 | 3 |

Only included fields that had ≥ 3 guidelines; AGREE: The Appraisal of Guidelines for Research & Evaluation; ^a^ Ranking based on mean overall assessment scores; ^b^ General fields (screening, diagnosis, et al.).
