# Supplementary material for: Quality of pediatric clinical practice guidelines
Source: BMC Pediatr. 2021 May 7;21:223. doi: 10.1186/s12887-021-02693-1 (PMC8103635; doi:10.1186/s12887-021-02693-1)
Supplement: Supplementary file 2 — Additional file 2: Supplemental Table 2. Comparison of standardized scores in each domain of guidelines established by different organizations or groups by AGREE II. [file 12887_2021_2693_MOESM2_ESM.docx]

**Online Only**

Supplemental Table 2. Comparison of standardized scores in each domain of guidelines established by different organizations or groups by AGREE II.

| Subject | Scope and purpose | Stakeholder involvement | Rigor of development | Clarity of presentation | Applicability | Editorial independence | Overall assessment | Ranking ^a^ |
| --- | --- | --- | --- | --- | --- | --- | --- | --- |
| Overall | 55.16% | 34.22% | 28.62% | 66.77% | 21.26% | 35.26% | 4.26 | - |
| Organization/ Group | | |  |  |  |  |  |  |
| AAP | 55.87% | 39.82% | 31.48% | 61.11% | 22.92% | 48.15% | 5.89 | 3 |
| ACR | 46.66% | 24.44% | 22.30% | 53.33% | 20.00% | 12.50% | 3.00 | 12 |
| CDC | 59.26% | 37.04% | 34.03% | 68.52% | 34.03% | 56.94% | 4.67 | 5 |
| EEEC | 35.47% | 20.51% | 29.89% | 73.72% | 11.22% | 22.44% | 4.23 | 7 |
| ESPGHAN | 57.41% | 33.33% | 47.22% | 73.15% | 22.92% | 38.89% | 4.67 | 6 |
| ICMR | 57.54% | 35.71% | 28.87% | 69.44% | 21.13% | 42.86% | 4.14 | 8 |
| ISPAD | 45.28% | 24.44% | 9.90% | 71.53% | 15.42% | 5.62% | 3.75 | 9 |
| NICE | 59.72% | 47.22% | 23.96% | 64.06% | 34.24% | 19.79% | 5.56 | 4 |
| None | 52.15% | 33.60% | 26.51% | 57.35% | 15.26% | 37.23% | 3.65 | 10 |
| QH | 71.87% | 56.60% | 52.35% | 79.86% | 57.29% | 66.67% | 6.00 | 2 |
| TNS | 58.49% | 23.45% | 12.33% | 71.91% | 16.78% | 41.44% | 3.44 | 11 |
| WHO | 78.71% | 65.28% | 59.38% | 84.72% | 47.22% | 63.89% | 6.83 | 1 |

Only included organizations or groups that had ≥ 3 guidelines; AGREE: The Appraisal of Guidelines for Research & Evaluation; AAP: American Academy of Pediatrics; ACR: American College of Radiology; CDC: Centers for Disease Control and Prevention (the U.S.); EEEC: ESPGHAN (European Society Paediatric Gastroenterology, Hepatology and Nutrition)/ ESPEN (The European Society for Clinical Nutrition and Metabolism)/ ESPR (European Society for Paediatric Research)/ CSPEN (Chinese Society for Parenteral and Enteral Nutrition) Working Group; ESPGHAN: European Society Paediatric Gastroenterology, Hepatology and Nutrition; ICMR: Indian Council of Medical Research; ISPAD: International Society for Pediatric and Adolescent Diabetes; NICE: National Institude for Health and Care Excellence (the U.K.); QCG: Queensland Health; TNS: Turkish Neonatal Society; WHO: World Health Organization; ^a^ Ranking based on mean overall assessment scores.
