# Supplementary material for: Quality of pediatric clinical practice guidelines
Source: BMC Pediatr. 2021 May 7;21:223. doi: 10.1186/s12887-021-02693-1 (PMC8103635; doi:10.1186/s12887-021-02693-1)
Supplement: Supplementary file 3 — Additional file 3: Supplemental Table 3. Comparison of standardized scores in each domain of guidelines established by different countries or regions by AGREE II. [file 12887_2021_2693_MOESM3_ESM.docx]

**Online Only**

Supplemental Table 3. Comparison of standardized scores in each domain of guidelines established by different countries or regions by AGREE II.

| Subject | Scope and purpose | Stakeholder involvement | Rigor of development | Clarity of presentation | Applicability | Editorial independence | Overall assessment | Ranking ^a^ |
| --- | --- | --- | --- | --- | --- | --- | --- | --- |
| Overall | 55.16% | 34.22% | 28.62% | 66.77% | 21.26% | 35.26% | 4.26 | - |
| Country/ Region | | |  |  |  |  |  |  |
| Australia | 63.49% | 49.21% | 43.53% | 70.04% | 38.99% | 54.76% | 5.00 | 2 |
| Canada | 57.41% | 31.94% | 45.14% | 74.54% | 20.14% | 38.89% | 4.50 | 4 |
| Cooperation | 52.11% | 32.26% | 28.53% | 68.32% | 19.27% | 31.38% | 4.24 | 5 |
| Germany | 50.00% | 26.67% | 16.67% | 57.22% | 17.08% | 40.00% | 3.40 | 9 |
| India | 58.95% | 38.27% | 22.92% | 61.42% | 18.75% | 48.61% | 3.78 | 7 |
| Italy | 64.58% | 44.44% | 54.43% | 77.78% | 25.52% | 47.92% | 5.00 | 3 |
| Turkey | 58.49% | 23.45% | 12.33% | 71.91% | 16.78% | 41.44% | 3.44 | 8 |
| The U.K. | 58.65% | 43.38% | 33.42% | 64.10% | 27.72% | 31.09% | 5.04 | 1 |
| The U.S. | 54.27% | 32.16% | 23.92% | 58.65% | 17.87% | 32.05% | 3.96 | 6 |

Only included countries or regions that had ≥ 3 guidelines; AGREE: The Appraisal of Guidelines for Research & Evaluation; ^a^ Ranking based on mean overall assessment scores.
